# Cultivar Contributes to the Beneficial Effects of *Bacillus subtilis* PTA-271 and *Trichoderma atroviride* SC1 to Protect Grapevine Against *Neofusicoccum parvum*

**DOI:** 10.3389/fmicb.2021.726132

**Published:** 2021-10-14

**Authors:** Catarina Leal, Nicolas Richet, Jean-François Guise, David Gramaje, Josep Armengol, Florence Fontaine, Patricia Trotel-Aziz

**Affiliations:** ^1^University of Reims Champagne-Ardenne, Résistance Induite et Bioprotection des Plantes Research Unit, EA 4707, INRAE USC 1488, SFR Condorcet FR CNRS 3417, Reims, France; ^2^Instituto de Ciencias de la Vid y del Vino, Consejo Superior de Investigaciones Científicas, Universidad de la Rioja, Gobierno de La Rioja, Logroño, Spain; ^3^Instituto Agroforestal Mediterráneo, Universitat Politècnica de València, Valencia, Spain

**Keywords:** Chardonnay, Tempranillo, biocontrol, *Neofusicoccum parvum*, plant immunity, synergistic effect

## Abstract

Grapevine trunk diseases (GTDs) are a big threat for global viticulture. Without effective chemicals, biocontrol strategies are developed as alternatives to better cope with environmental concerns. A combination of biological control agents (BCAs) could even improve sustainable disease management through complementary ways of protection. In this study, we evaluated the combination of *Bacillus subtilis* (*Bs*) PTA-271 and *Trichoderma atroviride* (*Ta*) SC1 for the protection of Chardonnay and Tempranillo rootlings against *Neofusicoccum parvum* Bt67, an aggressive pathogen associated to Botryosphaeria dieback (BD). Indirect benefits offered by each BCA and their combination were then characterized *in planta*, as well as their direct benefits *in vitro*. Results provide evidence that (1) the cultivar contributes to the beneficial effects of *Bs* PTA-271 and *Ta* SC1 against *N. parvum*, and that (2) the *in vitro* BCA mutual antagonism switches to the strongest fungistatic effect toward *Np*-Bt67 in a three-way confrontation test. We also report for the first time the beneficial potential of a combination of BCA against *Np*-Bt67 especially in Tempranillo. Our findings highlight a common feature for both cultivars: salicylic acid (SA)-dependent defenses were strongly decreased in plants protected by the BCA, in contrast with symptomatic ones. We thus suggest that (1) the high basal expression of SA-dependent defenses in Tempranillo explains its highest susceptibility to *N. parvum*, and that (2) the cultivar-specific responses to the beneficial *Bs* PTA-271 and *Ta* SC1 remain to be further investigated.

## Introduction

Global environmental changes promote the incidence of plant diseases by increasing the pathogen pressure or make the plants more susceptible to them (O’Brien, 2017; Velásquez et al., 2018). Grapevine trunk diseases (GTDs) are among the most important groups of grapevine diseases all over the world, creating a big concern in all wine-producing countries (Mondello et al., 2018). Attacking the plant perennial part and leading inevitably to the short- or long-term death of vines, the pathogens responsible of GTDs are described as very injurious for the sustainability of the winemaking industry (Hofstetter et al., 2012). As main restrictors for viticulture, GTDs can lead to high economic losses, less table grape for consumers, and social and environmental disturbances (O’Brien, 2017). Both young and mature vines are affected by GTDs, even as nursery staged plants, reducing both productivity and longevity of the vineyard, thereby causing massive economic losses (Gramaje and Armengol, 2011).

Botryosphaeria dieback (BD) is one of the most significant GTDs, triggerable by more than 25 distinct species of Botryosphaeriaceae including the aggressive *Neofusicoccum parvum* (Úrbez-Torres, 2011; Billones-Baaijens and Savocchia, 2019; Reis et al., 2019; Larach et al., 2020). Symptomatic plants develop a low or apoplectic dieback phenotype, including a low budburst rate, a poor vegetative development, external canker, and internal longitudinal necrotic lesions that can lead to a full dead branch (Larignon et al., 2001, 2009; Larignon, 2004; Billones-Baaijens and Savocchia, 2019; Larach et al., 2020). Susceptibility to BD pathogens also differs between cultivars (Travadon et al., 2013; Fontaine et al., 2016a; Chacon et al., 2020; Claverie et al., 2020).

Due to the undetermined period of latency within the vines (asymptomatic state), early detection and management of GTDs remain presently a challenge in both nursery and vineyard, and only few preventives, but no curative methods, are available. Indeed, few chemicals are applied on pruning wounds in vineyards to prevent dissemination of the conidia of fungal pathogens (Sosnowski et al., 2004), preferring cultural practices in vineyard (Mondello et al., 2018) and sanitation methods (Gramaje and Armengol, 2011; Gramaje et al., 2018). However, these kinds of treatments cannot eradicate the pathogens once established in a vineyard (Calzarano et al., 2004; Gramaje and Armengol, 2011). An interesting alternative and complement to the previously cited GTDs control methods in vineyard is the use of biological control agents (BCAs) as reported by Mondello et al. (2018). For instance, several microorganisms have already been evaluated, both *in vitro* and *in planta* against BD pathogens (Hunt et al., 2001; Di Marco et al., 2004; John et al., 2004; Compant et al., 2013; Compant and Mathieu, 2017; Trotel-Aziz et al., 2019). Among them there are bacterial BCAs such as *Pseudomonas*, *Bacillus*, and *Enterobacter* species, and fungal BCAs such as several *Fusarium* and *Trichoderma* species (Mondello et al., 2018). Some of them, such as *Trichoderma atroviride*, *Trichoderma harzianum*, *Bacillus subtilis*, and *Bacillus amyloliquefaciens*, are already commercialized against some GTDs pathogens, or against other hemibiotrophic and necrotrophic pathogens including *Botrytis cinerea*, *Fusarium oxysporum*, or many others (Elad, 2000; Schmidt and Panstruga, 2011; Kuzmanovska et al., 2018; Thambugala et al., 2020; Alfiky and Weisskopf, 2021).

To date, *Trichoderma* species are the most used fungal-based BCA in viticulture (Harman, 2006; Muckherjee et al., 2012; Waghunde et al., 2016) and have been also widely investigated as BCA against GTDs (Di Marco et al., 2002, 2004; John et al., 2008; Halleen et al., 2010: Berbegal et al., 2020; Martínez-Diz et al., 2021a,b). *Trichoderma* spp. are described to directly antagonize GTD pathogen aggressiveness by competition for nutrients and space, mycoparasitism, cell-wall degrading enzymes, and antibiosis (Harman, 2006; Van Wees et al., 2008; Vinale et al., 2008; Pieterse et al., 2014; Waghunde et al., 2016). *Trichoderma* spp. have also been described as plant growth and defense stimulators (Harman, 2006; Van Wees et al., 2008; Vinale et al., 2008; Pieterse et al., 2014; Waghunde et al., 2016). Among *Trichoderma* spp., *T. atroviride* SC1 was shown to strongly reduce the infections caused by some GTD pathogens in nurseries and established vineyards at the registered dose rate of 2 g/L, equivalent to the density of 2 × 10^10^ conidia/L recommended by the commercial product (Pertot et al., 2016; Berbegal et al., 2020; Martínez-Diz et al., 2021a). *Bacillus* strains are another group of microorganisms extensively studied as BCA and reported to directly and indirectly protect plants against pathogens with different lifestyles (Magnin-Robert et al., 2007; Trotel-Aziz et al., 2008; Nguyen et al., 2020), including the GTD hemibiotrophic pathogens (Schmidt et al., 2001; Halleen et al., 2010; Rezgui et al., 2016; Kotze et al., 2019; Trotel-Aziz et al., 2019). A broad range of beneficial molecules are produced or encoded by the genome of *Bacillus* spp., both to induce or elicit plant defenses (as with phytohormones precursors, lipopolysaccharides, siderophores, etc.) and to directly compete, antagonize, or alter plant pathogens or their aggressiveness (Kloepper et al., 2004; Ongena and Jacques, 2008; Leal et al., 2021). Among *Bacillus* spp., *B. subtilis* is one of the most frequently tested against GTDs (Mondello et al., 2018), and *B. subtilis* PTA-271 has shown promising results in reducing infections caused by the aggressive strain *N. parvum* Bt67 (Trotel-Aziz et al., 2019). Literature also reports that the combination of two or more BCAs can improve the management of plant diseases (Weller, 1988; Guetsky et al., 2002; El-Tarabily, 2006; Yobo et al., 2011; Magnin-Robert et al., 2013), probably due to additive or synergistic effects of combined mechanisms in a complex changing environment (Meyer and Roberts, 2002).

Beneficial microbial interactions conferred by BCA lead to induced systemic resistance (ISR) in the plant, giving it greater protection to pathogens in spatially separated parts of the plant (Alström, 1991; Van Peer et al., 1991; Wei et al., 1991; De Vleesschauwer and Höfte, 2009). ISR is associated with an early, strong, and rapid activation of plant defenses upon pathogen infection, a phenomenon known as the priming state (Conrath et al., 2001, 2015; Pieterse et al., 2014). Among the BCA-induced defense responses, the most relevant are jasmonate (JA)- and ethylene (ET)-dependent ones, described as useful defenses against necrotrophs (Pieterse et al., 2001, 2014; Verhagen et al., 2004; Van der Ent et al., 2009; Niu et al., 2011; Nie et al., 2017). However, Niu et al. (2011) also reported that some BCAs may mediate ISR in a salicylic acid (SA)-dependent manner. In brief, the diversity of BCA ways of protection may depend on both the BCA and the pathogen, but also on the plant and even the cultivar (Mutawila et al., 2011; Pacifico et al., 2019; Nguyen et al., 2020; Stempien et al., 2020).

In this study, we evaluated the effect of combining a potential BCA, *B. subtilis* PTA-271 (thereafter *Bs* PTA-271), and a BCA-commercial product containing *T. atroviride* SC1 (thereafter *Ta* SC1), on the protection of two distinct grapevine cultivars, Chardonnay and Tempranillo, potentially showing distinct susceptibilities to GTDs. The pathogen selected was *N. parvum* Bt67 (thereafter *Np*-Bt67), described as a very aggressive pathogen associated to BD. As each BCA has already been recognized as beneficial to at least one cultivar (Trotel-Aziz et al., 2019; Berbegal et al., 2020; Martínez-Diz et al., 2021a), their beneficial effect was additionally investigated on the other cultivar, as single BCA and in dual combination of two BCAs. To compare the two BCAs, densities were aligned to the density optimized for *Bs* PTA-271 with Chardonnay rootlings. After looking for the protective capacity of BCAs *in planta*, their ways of action leading to protection were further explored. Thus, the indirect and direct benefits offered by each BCA and their combination were investigated, focusing on both grapevine immunity and the direct beneficial or detrimental physical interplays among the microorganisms *in vitro* (*Bs* PTA-271, *Ta* SC1, and *Np*-Bt67).

## Materials and Methods

### Plant Material and Growth Conditions

Three-node-long cuttings of grapevine *Vitis vinifera* L. cv. Tempranillo (clone RJ-26) and cv Chardonnay (clone 7535) were provided by Viveros Villanueva nursery (Navarra, Spain) and Pommery’s vineyards in Reims (France), respectively. Tempranillo cuttings were surface-sterilized for 6 h in a 0.05% cryptonol (8-hydroxyquinoline sulfate) solution, waxed and stored at 4°C in a cold chamber for 3 weeks, and then rehydrated with 0.05% cryptonol solution overnight. Chardonnay cuttings were directly surface-sterilized with 0.05% cryptonol solution overnight. Cuttings of both cv were then rooted as described by Lebon et al. (2005), using an indole-3-butyric acid (1 g/L) solution before being placed by 15 in 350-ml pots containing the soil Sorexto (horticultural soil M4600, Grenoble, France) in a culture chamber (24/20°C day/night, 55–65% relative humidity day/night, and 16-h photoperiod at 400 μmol/m^2^/s). They were watered three times a week. Only rootlings that have developed roots (30% rooting rate in 15 weeks) were kept for further experiments and transferred to individual 200-ml pots with the same culture conditions.

### Biocontrol Agent’s Growth and Plant Treatments

#### *Bacillus subtilis* PTA-271

**FIGURE 1:**
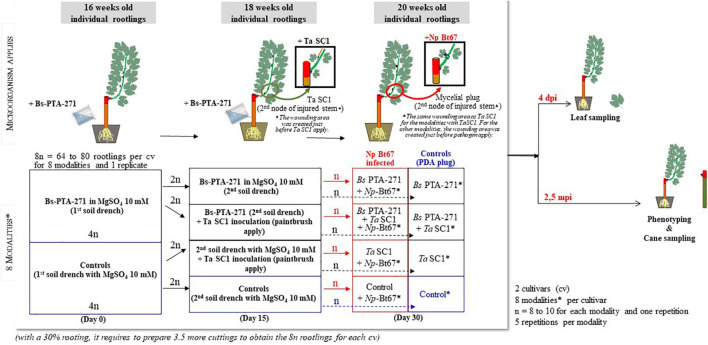
Diagram showing the *Bacillus subtilis* PTA-271 and *Trichoderma atroviride* SC1 inoculations, *Neofusicoccum parvum* strain Np-Bt67 infection, and sample collection process. *Modalities: Control, Bs PTA-271, Ta SC1, Bs PTA-271 + Ta SC1, Control + Np Bt67, Bs PTA-271 + Np Bt67, Ta SC1 + Np Bt67, Bs PTA-271 + Ta SC1 + Np Bt67.

*Bacillus subtilis* PTA-271 (GenBank Nucleotide EMBL Accession No. AM293677 for 16S rRNA and DDBJ/ENA/GenBank Accession No. JACERQ000000000 for the whole genome) was isolated in 2001 from the rhizosphere of healthy Chardonnay grapevines (*V. vinifera* L. cv. Chardonnay) from a vineyard located in Champagne (Marne, France) (Trotel-Aziz et al., 2008; Leal et al., 2021). Bacterial growth started by adding 100 μl of glycerol stock suspension to sterile Luria Bertani (LB) medium and incubating at 28°C with agitation (100 rpm). Experiments were performed when the bacterial culture is at the exponential growth phase. After centrifugation (5,000 *g*, 10 min), the pellet was washed once with a sterile 10 mM MgSO_4_ medium and resuspended in a same MgSO_4_ medium. Bacterial density was measured by spectrophotometry at 450 and 650 nm, and the mean density was adjusted with a sterile MgSO_4_ medium before treatment according to Trotel-Aziz et al. (2019). The bacterial suspension was applied twice by drenching the soil at the root level of rootlings at a final density of 10^8^ CFU/g soil. Inoculations were carried out when rootlings were 16 weeks old (considered as day 0) and 2 weeks later (day 15) as indicated in Figure 1. Control rootlings were similarly drenched twice with MgSO_4_ solution (Figure 1).

#### *Trichoderma atroviride* SC1

*Trichoderma atroviride* SC1 (Vintec^®^, Belchim Crop Protection, Bi-PA; 10^10^ conidia per gram of formulated product) was suspended in water at 10^8^ CFU/ml to compare the effect of each BCA at an equal density. In order to also take advantage of an eliciting effect, the *Ta* SC1 fungal suspension was applied once with a paintbrush to the second node of the previously wounded lignifying stem (5 mm diameter and 1 mm deep; made just before *Ta* SC1 apply) of 18-week-old rootlings (Figure 1). The inoculation site was immediately covered with parafilm (day 15).

### Pathogen Strain and Growth

*Neofusicoccum parvum* strain Bt67 isolated from Portuguese vineyards (Estremadura region) is inscribed in the HIA collection (Lisbon University, Portugal). This fungus was maintained on potato dextrose agar (PDA, Sigma, Saint-Quentin-Fallavier, France) plates and stored at 4°C (Trotel-Aziz et al., 2019). The resulting mycelium was plated on PDA medium and incubated in the dark at 22°C for 7 days before inoculation.

### Pathogen Inoculation to Plants, Quantification of Disease Symptoms, and Re-isolation of the Pathogen

**FIGURE 2:**
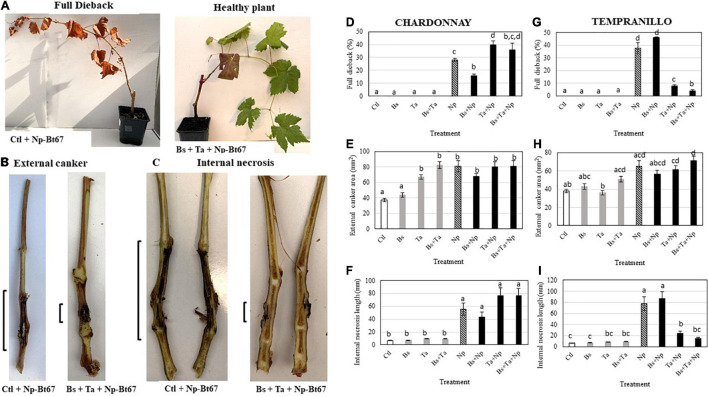
The beneficial combination of *Bacillus subtilis* PTA-271 and *Trichoderma atroviride* SC1 attenuates the Botryosphaeriaceae dieback symptoms induced in Chardonnay **(D–F)** and Tempranillo **(G–I)** rootlings by the *Neofusicoccum parvum* strain Np-Bt67. One-month pretreated grapevine rootlings with PTA-271, *Ta* SC1, and both BCA (Bs, Ta, and Bs + Ta) and non-treat plants (Ctl) were inoculated with pathogen mycelium (Np). Non-infected plants were inoculated with sterile medium without pathogen (Control). Compared to healthy asymptomatic rootlings (**A**, right), the infected symptomatic rootlings showed the typical Botryosphaeria dieback symptoms: full dieback percentage (**A** left, **E**), stem external canker **(B,E,H)** and stem internal necrosis **(C,F,I)** that were photographed **(A–C)** and quantified **(D–I)** at 2.5 months post-inoculation. Data are means ± standard deviation (SD) for at least three independent experiments with 10 biological replicates per treatment. Vertical bars with different letters are significantly different (multiple comparison procedures with Tukey’s test, *p* < 0.05).

Half of the 20-week-old rootlings that were treated with *Bs* PTA-271 (day 0 and day 15) and/or *Ta* SC1 (day 15) were further infected with the pathogen at the wounding area with a 3-mm-diameter mycelial plug from a 7-day-old culture of *Np*-Bt67 strain (day 30), thus at distinct time points (days 0–30) as summarized in Figure 1. The inoculation site was then covered with moist hydrophilic cotton before sealing with parafilm. The experiment was composed of five repetitions for each modality (treatment), with 8–10 replicate plants per treatment (Figure 1). To confirm that lesions were due to pathogen infection and not to the injury, the control plants were also injured and inoculated with sterile 3-mm PDA plugs (Figure 1). Rootlings, namely, “control,” are those that are neither treated with BCA nor infected with the pathogen. After inoculation, vines were kept in the same culture chamber and BD symptoms were assessed at 2.5 months post-inoculation (mpi) (Figure 1). Disease symptoms were evaluated as described by Trotel-Aziz et al. (2019) by quantifying the percentage of the full dead shoots from inoculated rootlings (Figure 2A, “Full dieback”) and by measuring both the external canker area and the internal necrosis length of the other lignified shoots (Figures 2B,C, respectively). To check the success of the infection and the lack of contaminations, re-isolation of pathogen was performed as described by Pinto et al. (2018), by quickly passing the infected stems onto the flame, then removing the top of the necrotic zone with a scalpel, before plating seven small pieces of tissue per plant onto PDA plates. Plates were then incubated at 28°C for at least 7 days. For every repetition of the experiments, re-isolations were performed with seven of the infected rootlings per infected combination (i.e., Np, Bs + Np, Ta + Np, and Bs + Ta + Np) and each replicate, and one negative control rootling per non-infected combination (i.e., Control, Bs, Ta, and Bs + Ta).

### Direct Confrontation Tests With Biocontrol Agents and Pathogen

Antagonism among microorganisms was checked *in vitro*, using *B. subtilis* PTA-271, *T. atroviride* SC1, and *N. parvum Np*-Bt67, in dual or three-way confrontation tests on PDA plates (9 mm diameter). *Bs* PTA-271 grown in LB medium and *Ta* SC1 resuspended in sterilized water were both used at 10^8^ CFU/ml (5 μl drop), while a 7-day-old mycelium plug (3 mm) was used for the pathogen. Three types of direct confrontation were performed: (A) the pathogen/BCA combinations were plated at the same time, but at distinct areas (i.e., 5 cm away from each other); (B) one or two isolates (*Bs* PTA-271, *Ta* SC1, or *Np*-Bt67) were plated 48 h before the other(s), and at distinct areas; and (C) isolates were plated simultaneously and at the center of the plate. Controls containing one single isolate (*Bs* PTA-271, *Ta* SC1, or *Np*-Bt67) were also made, either on the side of the plate or at the center. All plates were incubated in the dark at 28°C for at least 11 days and photographed daily. Antagonistic effect was characterized by an inhibition zone around the BCA and/or the pathogen. Since the first kind of confrontation (A) added no more information compared to the others (B and C), it was not shown in this study. The experiment was conducted twice with three replicate plates per treatment, and the area occupied by each microorganism was measured daily using ImageJ software (Rueden et al., 2017), based on a reference distance common to all images.

### RNA Extraction and Quantitative Reverse-Transcription Polymerase Chain Reaction Analysis

From the *in planta* assays with rootlings, leaf samples were collected 4 days post-inoculation of pathogen (dpi), ground in liquid nitrogen, and then stored at −80°C. RNA was extracted from powdered 40 leaves of 8 rootlings per replicate of each modality. Total RNA was extracted from 50 mg of leaf powder with Plant RNA Purification Reagent according to manufacturer instructions (Invitrogen, Pontoise, France), and DNase treated as described by the manufacturer’s instructions (RQ1 RNase-Free DNase, Promega). RNA quality was checked by agarose gel electrophoresis, and total RNA concentration was measured at 260 nm for each sample using the NanoDrop One spectrophotometer (Ozyme) and adjusted to 100 ng μl^–1^. First-strand cDNA was synthesized from 150 ng of total RNA using the Verso cDNA synthesis kit (Thermo Fisher Scientific, Inc., Waltham, MA, United States). Polymerase chain reaction (PCR) conditions were the ones described by Gruau et al. (2015). Quantitative reverse-transcription polymerase chain reaction (qRT-PCR) was performed with Absolute Blue qPCR SYBR Green ROX Mix according to manufacturer instructions (Thermo Fisher Scientific, Inc., Waltham, MA, United States), in a BioRad C1000 thermocycler using the Bio-Rad manager software CFX96 Real-Time PCR (BioRad, Hercules, CA, United States). A set of six defense-related genes, selected for their responsiveness to pathogen or priming state induced by beneficial microorganisms (Trotel-Aziz et al., 2019), was tracked by qRT-PCR using specific primers (Table 1). Quantitative RT-PCR reactions were carried out in duplicate in 96-well plates in a 15-μl final volume containing Absolute Blue SYBR Green ROX mix including Taq polymerase ThermoPrime, dNTPs, buffer, and MgCl_2_ (Thermo Fisher Scientific, Inc., Waltham, MA, United States), 280 nM forward and reverse primers, and 10-fold diluted cDNA according to the manufacturer’s protocol. Cycling parameters were 15 min of Taq polymerase activation at 95°C, followed by 40 two-step cycles composed of 10 s of denaturation at 95°C and 45 s of annealing and elongation at 60°C. Melting curve assays were performed from 65 to 95°C at 0.5°C s^–1^, and melting peaks were visualized to check amplification specificity. EF1 and 60SRP genes were used as references and experiments were repeated five times. Relative gene expression was determined with the formula fold induction: 2(−11Ct), where 11Ct = [Ct TG (US) − Ct RG (US)] − [Ct TG (RS) − Ct RG (RS)], where Ct is cycle threshold, Ct value is based on the threshold crossing point of individual fluorescence traces of each sample, TG is target gene, RG is reference gene, the US is an unknown sample, and RS is reference sample. Integration of the formula was performed by the CFX Manager 3.1 software (Bio-Rad). Although the genes analyzed were considered significantly up- or downregulated when changes in their expression were >2-fold or <0.5-fold, respectively, we still performed a statistical analysis. Control samples for the rootlings model are cDNA from leaves of rootlings untreated with BCA and inoculated with sterile PDA plugs (1× expression level).

**TABLE 1:** Primer sequences used for qRT-PCR analysis of defense-related genes (Trotel-Aziz et al., 2019).

Gene	Name	Accession number^1^	Forward primer (5′-3′)	Reverse primer (5′-3′)	Annealing temperature (°C)	Amplicon size (bp)	Efficiency of primers pairs (%)
*60RSP*	60S ribosomal protein L18	XM_002270599 ^1^	ATCTACCTCAAGCTCCTAGTC	CAATCTTGTCCTCCTTTCCT	60	166	100.0
*EF1*	Elongation factor 1-alpha	XM_002284888 ^1^	AACCAAAATATCCGGAGTAA AAGA	GAACTGGGTGCTTGATAGGC	60	164	100.0
*LOX9*	Lipoxygenase	NM_001281249 ^1^	CCCTTCTTGGCATCTCCCTTA	TGTTGTGTCCAGGGTCCATTC	60	101	90.0
*PR1*	Pathogenesis- related protein 1	XM_002273752 ^1^	GGAGTCCATTAGCACTCCTTTG	CATAATTCTGGGCGTAGGCAG	60	168	90.0
*PR2*	Class I beta-1,3-glucanase	NM_0012809671	TCAATGGCTGCAATGGTGC	CGGTCGATGTTGCGAGATTTA	60	155	97.2
*GST1*	Glutathione-*S*-transferase	NM_001281248 ^1^	TGCATGGAGGAGGAGTTCGT	CAAGGCTATATCCCCATTTTCTTC	60	98	90.0
*PAL*	Phenylalanine ammonia lyase	XM_003635637 ^1^	TCCTCCCGGAAAACAGCTG	TCCTCCAAATGCCTCAAATCA	60	101	92.9
*STS*	Stilbene synthase	NM_001281117 ^1^	AGGAAGCAGCATTGAAGGCTC	TGCACCAGGCATTTCTACACC	60	101	94.3

*^1^NCBI accession number.*

### Statistical Analysis

Data of canker area and length of external and internal necrosis of the stems were obtained by the analysis of photos using ImageJ software (Rueden et al., 2017). Statistical analyses were carried out using all the vines of three replicates among five for each modality with RStudio software (Horton and Kleinman, 2015). For modality significance, mean values were compared by Tukey’s test (*p* < 0.05). Results of confrontation tests are from one representative repetition out of two showing the same trends. Statistical analyses were carried out using the SigmaStat 3.5 software. For treatment effect, mean values were compared by Tukey’s test (*p* < 0.05). Results of gene expression by qRT-PCR analysis correspond to means ± SEM deviation from three representative repetitions out of five showing the same trends. Statistical analyses were carried out using the XLSTAT 2021.1.1 5 software (Addinsoft, Paris, France). For treatment effect, mean values were analyzed using one-way analysis of variance (ANOVA). When differences in the means were significant, Fisher’s LSD *post hoc* test (α = 0.1) was applied to determine which treatments were significantly different from others.

## Results

### Effects of *Bacillus subtilis* PTA-271 and *Trichoderma atroviride* SC1 on Two Cultivars Infected With *Neofusicoccum parvum* Bt67

In control Chardonnay infected with the pathogen, the results of infection showed a rate of 28 ± 1.24% for dead shoot (full dieback), of 80.6 ± 7.35 mm^2^ for external canker size, and of 55.4 ± 9.44 mm for internal necrosis length (see *Np*-Bt67 in Figures 2D–F), while in Tempranillo, they reached 37.5 ± 4.56%, 65.2 ± 6.23 mm^2^, and of 77.8 ± 11.95 mm, respectively (see *Np*-Bt67 in Figures 2G–I).

In BCA-treated Chardonnay rootlings then infected with the pathogen, the results of the biocontrol assays showed that *Bs*-PTA-271-pretreated plants presented a significant lower number of plants with full dieback (by approximately 45%) than the infected control (Figure 2D). Infected plants pretreated with *Ta* SC1 did not reduce the full dieback development compared to infected control plants, while infected plants pretreated with both BCAs showed a great variability in the development of full dieback symptoms. Similarly, the external canker area (Figure 2E) and the internal necrosis length of infected Chardonnay (Figure 2F) were solely slightly reduced in *Bs*-PTA-271-pretreated rootlings (by 16 and 22%, respectively), but insignificantly (Figure 2E). In contrast, necrosis length was increased in *Ta* SC1 and both BCAs pretreated plants compared to infected control, although non-significant (Figure 2F).

In BCA-treated Tempranillo rootlings then infected with the pathogen, the results of the biocontrol assays showed that *Ta*-SC1- and combined-BCA-pretreated plants showed a significant lower number of full dieback (by approximately 80 and 91%, respectively) and length of stem internal necrosis (by approximately 70 and 81%, respectively) than the infected control (Figures 2G,I, respectively). In contrast, infected plants pretreated with *Bs* PTA-271 showed that neither full dieback development nor internal stem necrosis reduced, compared to infected control plants. Looking at the external canker area (Figure 2H), none of the treatments with *Bs*-PTA- 271-, *Ta*- SC1-, and combined-BCA-pretreated plants, consecutively infected, induced any significant difference with the infected control. Therefore, external canker may not appear as a relevant indicator for Np dieback with this experimental model, for both Tempranillo and Chardonnay.

Re-isolations of the pathogen confirmed that (1) there was no background infection elsewhere than in the artificially infected rootlings, satisfying thus the Koch’s postulates, and that (2) the pathogen was still alive in both dead and living stems of plants, as well as in infected plants pretreated or not with BCA. From all infected plants, the pathogen was successfully isolated with a percentage of success >90% that indicated no fungicidal effect from BCA toward *Np*-Bt67.

### Effects of Biological Control Agents on the Basal Defense of Chardonnay and Tempranillo

The ability of *Bs* PTA-271 or *Ta* SC1 or both BCAs to enhance grapevine immunity was addressed in leaves of control rootlings. Six selected defense genes were targeted by qRT-PCR: the lipoxygenase *LOX9* involved in oxylipin synthesis and described as dependent to JA/ET (Hamiduzzaman et al., 2005; Naznin et al., 2014); *PR1* described to be regulated by SA(Dufour et al., 2013; Naznin et al., 2014; Caarls et al., 2015); the β-1,3-glucanase *PR2* described to be regulated by various phytohormones such as SA, JA, and ET (Liu et al., 2010); the glutathione-*S*-transferase *GST1* putatively involved in the detoxification process; the phenylalanine ammonia-lyase *PAL* catalyzing the first step in the phenylpropanoid pathway; and the stilbene synthase *STS* involved in the synthesis of phytoalexins. Since BCAs were not detected in leaves (not shown) where defenses were induced, the induction of plant defense by BCAs is systemic.

**FIGURE 3:**
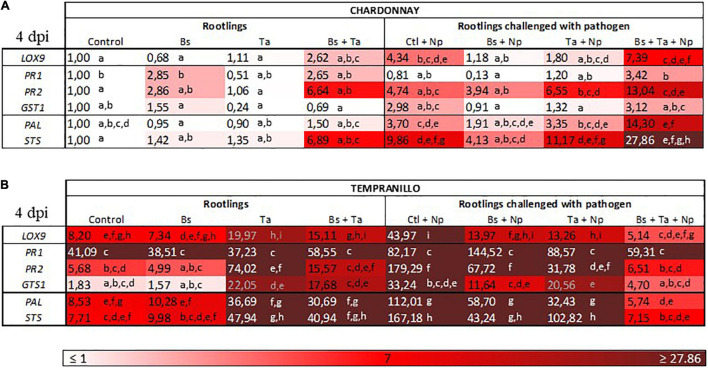
*Bacillus subtilis* PTA-271 and *Trichoderma atroviride* SC1 attenuates induced differential expression of defense-related genes in leaves of Chardonnay **(A)** and Tempranillo **(B)** rootlings before and after pathogen challenge. Twenty-week-old rootlings untreated or pretreated with PTA-271 or SC1 or both were further infected with sterile PDA plugs (Control, Bs, Ta, and Bs + Ta, respectively) or with mycelium plugs of Np-Bt67 (Ctl + Np, Bs + Np, Ta + Np, and Bs + Ta + Np, respectively). Transcript levels of defense-related genes monitored by qRT-PCR in plant leaves after 4 days of inoculation. Uninfected control of Chardonnay was considered as a reference sample (1× expression level) for both cultivars, and heatmaps represent changes in the transcript expression levels as indicated by the color shading. Data are the means from three representative replicates among five showing the same trends. Different letters indicate statistically significant differences between the treatments (ANOVA, Fisher’s LSD *post hoc* test, α = 0.1). Legends for genes are LOX9, lipoxygenase 9; PR1, pathogenesis-related protein 1; PR2, class I β-1,3-glucanase; GST1, glutathione-*S*-transferase 1; PAL, phenylalanine ammonia lyase; STS, stilbene synthase.

**FIGURE 4:**
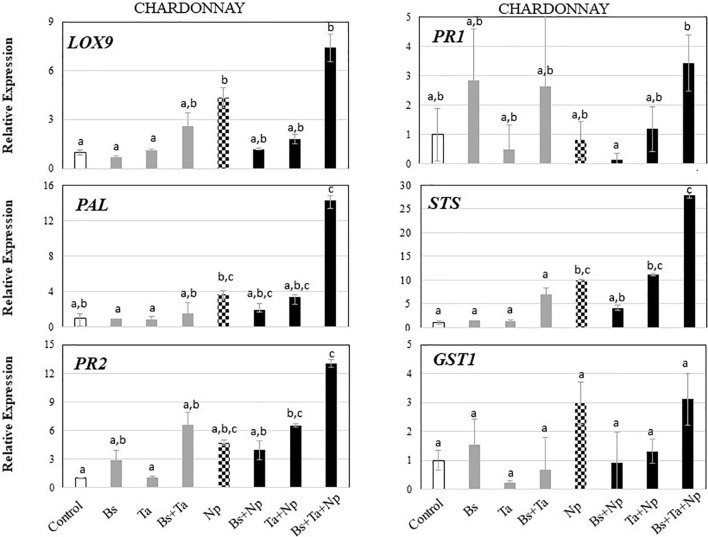
*Bacillus subtilis* PTA-271 and *Trichoderma atroviride* SC1 attenuates induced differential expression of defense-related genes in leaves of Chardonnay rootlings before and after pathogen challenge. Twenty-week-old rootlings untreated or pretreated with PTA-271 or SC1 or both were further infected with sterile PDA plugs (Control, Bs, Ta, and Bs + Ta, respectively) or with mycelium plugs of Np (Ctl + Np, Bs + Np, Ta + Np, and Bs + Ta + Np, respectively). Transcript levels of defense-related genes monitored by qRT-PCR in plant leaves after 4 days of inoculation. Uninfected control was considered as the reference sample (1× expression level). Data are the means from three representative replicates among five showing the same trends. Different letters indicate statistically significant differences between the treatments (ANOVA, Fisher’s LSD *post hoc* test, α = 0.1). Legends for genes are as in Figure 2.

Data showed some differences in the level of expression of the basal defense genes between the greenhouse cultivars (Figure 3), despite the fact that rootlings all grew in the same chamber of the greenhouse with similar culture conditions. The cultivar Chardonnay exhibited a weak constitutive expression of targeted defense genes (Figure 3A) compared to Tempranillo (Figure 3B). In Chardonnay (Figure 4 and Supplementary Figure 1A), the application of *Bs* PTA-271 at root level induced a 2.8-fold increase of *PR1* and *PR2* expression in leaves, while the application of *Ta* SC1 at stem level did not induce any consistent changes in the expression of these same defense genes in leaves. Interestingly, the application of both BCAs induced the expression of the greatest number of targeted genes in the leaves: a 2.6-fold expression of *LOX9* and a 6.6- or 6.8-fold expression of *PR2* and *STS*, respectively. *Bs* PTA-271, alone or together with *Ta* SC1, may thus act as a priming stimulus for Chardonnay cultivar.

**FIGURE 5:**
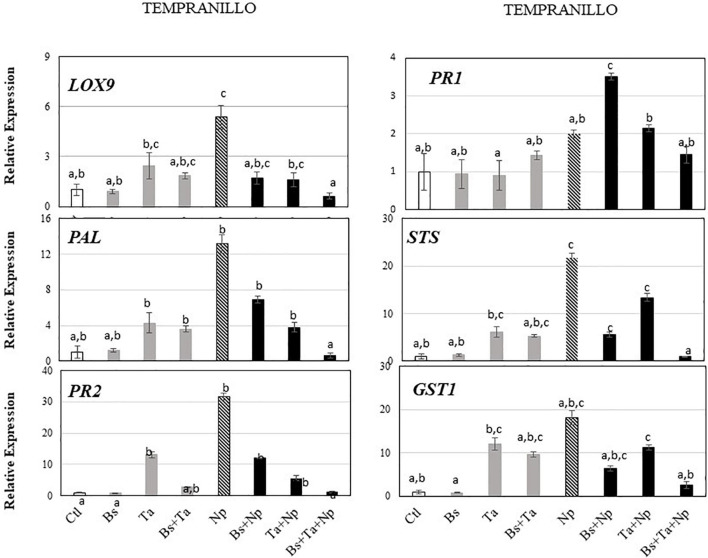
*Bacillus subtilis* PTA-271 and *Trichoderma atroviride* SC1 attenuates induced differential expression of defense-related genes in leaves of Tempranillo rootlings before and after pathogen challenge. Twenty-week-old rootlings untreated or pretreated with PTA-271 or SC1 or both were further infected with sterile PDA plugs (Control, Bs, Ta, and Bs + Ta, respectively) or with mycelium plugs of *Np*-Bt67 (Ctl + Np, Bs + Np, Ta + Np, and Bs + Ta + Np, respectively). Transcript levels of defense-related genes monitored by qRT-PCR in plant leaves after 4 days of inoculation. Uninfected control was considered as the reference sample (1× expression level). Data are the means from three representative replicates among five showing the same trends. Different letters indicate statistically significant differences between the treatments (ANOVA, Fisher’s LSD *post hoc* test, α = 0.1). Legends for genes are as in Figure 2.

Tempranillo (Figure 3B) showed a high basal expression of the targeted gene responsive to SA (i.e., *PR1*) compared to Chardonnay (Figure 3A). Interestingly, while the application of *Bs* PTA-271 (Figure 5 and Supplementary Figure 1B) did not induce any consistent changes of defense gene expression, that of *Ta* SC1 induced the expression of the greatest number of studied genes: by a factor of 2.4 for *LOX9*, 13.0 for *PR2*, 12.0 for *GST1*, 4.3 for *PAL*, and 6.2 for *STS*. To contrast with Chardonnay, no relevant number of targeted genes were overexpressed with the application of both BCAs (2.7-fold for *PR2*, 9.6-fold for *GST1*, 3.6-fold for *PAL*, and 5.3-fold for *STS*) compared to *Ta* SC1 alone. *Ta* SC1, alone or together with *Bs* PTA-271, may thus act as a priming stimulus for Tempranillo.

### Effects of Biological Control Agents on the Induced Defense of Chardonnay and Tempranillo Upon Pathogen Challenge

In leaves of Chardonnay rootlings infected with Np-Bt67, data from qRT-PCR showed that except for *PR1* (0.8-fold expression), the expression of all targeted defense genes was consistently upregulated from 2.9- to 9.8-fold (4.3 for *LOX9*, 4.7 for *PR2*, 2.9 for *GST1*, 3.7 for *PAL*, and 9.8 for *STS*) (Figure 4 and Supplementary Figure 1A). As indicated before, in the absence of pathogen infection, *Bs* PTA-271 only induced a weak expression of *PR2* and *PR1* (2.8-fold increase) but was not significant according to ANOVA analysis (Figure 4 and Supplementary Figure 1A), suggesting that *Bs* PTA-271 may act as a priming stimulus in Chardonnay. However, upon pathogen challenge, no post-priming was observed in *Bs*-PTA-271-pretreated plants since it did not induce any stronger activation of the targeted plant immune defenses compared to Chardonnay-infected control at 4 dpi (Figure 3). In contrast, *Ta* SC1 showed no sign of priming stimulus in control Chardonnay, but it induced similar expression of *PR2* (6.5-fold increase) and *STS* (11.1-fold increase) than infected control (4.7 and 9.8, respectively). Interestingly, the application of both BCAs enabled to reach the highest level of Chardonnay defense gene expression upon pathogen challenge (3.1–27.8) compared to infected control (0.8–9.8), a priming effect shown as consistent according to the discriminating capacity of the qRT-PCR technique, but still not yet significant according to the ANOVA analysis. Anyway, such a synergy at 4 dpi can result from a priming stimulus by *Bs* PTA-271, followed by a post-primed phase upon pathogen inoculation with a more rapid and strong activation of immune defenses due to interactions among each actor (*Bs* PTA-271, *Ta* SC1, *Np*-Bt67, and Chardonnay).

In leaves of Tempranillo infected with *Np*-Bt67, the expression of all targeted defense genes was consistently upregulated from 2.0- to 31.5-fold, and significantly for *LOX9*, *PR2*, *PAL*, and *STS* (Figure 5 and Supplementary Figure 1B). Compared to infected Chardonnay (Figure 3A), expression of the basal defense genes was significantly stronger in infected Tempranillo (Figure 3B), highlighting a higher basal defense level in Tempranillo toward *Np-*Bt67 than in Chardonnay at 4 dpi. The ability of each BCA or both to enhance Tempranillo immunity was also addressed (Figure 5 and Supplementary Figure 1B). As reported above, in the absence of pathogen infection, *Ta* SC1 alone induced a consistent expression of almost all the targeted defenses (2.44–13.03 for *LOX9*, *PR2*, *GST1*, *PAL*, and *STS*), suggesting that *Ta* SC1 may act as a priming stimulus for Tempranillo cultivar, but in a lesser extent when combined with *Bs* PTA-271 (1.84–9.66). However, upon pathogen challenge, no post-priming was observed in *Ta* SC1-pretreated rootlings since it did not induce any stronger expression of the targeted immune defenses than in the Tempranillo-infected control at 4 dpi, but lower (Figure 5 and Supplementary Figure 1B). Regarding *Bs* PTA-271 effect (i.e., Bs + Np), while it showed no sign of priming stimulus in control Tempranillo, it induced the expression of almost all targeted defenses, but similarly to *Ta* SC1, thus in a lower extent than the infected control (thus, no more priming in that condition with *Bs* PTA-271). Additionally, the pretreatment with both BCAs induced a lower expression level of Tempranillo defense genes upon pathogen challenge (0.63–2.57) than in infected control (2.0–31.56). However, such apparent non-expression of Tempranillo defenses at 4 dpi did not presume any useful induced defenses at other key times or among other defenses that were not targeted in this study.

Taking the uninfected control of Chardonnay as the reference sample for both cultivars, we can compare the immunity between Tempranillo and Chardonnay upon pathogen challenge (Figures 3A,B, respectively). As observed in control condition (i.e., high basal expression of *PR1*), infected Tempranillo showed once again a high expression of this targeted gene presumably responsive to SA, compared to Chardonnay. This suggests that Tempranillo would use SA-dependent defense pathways toward *N. parvum*, even when overexpressing *PR2*, *GST1*, *PAL*, and *STS*, unlike Chardonnay. These data thus highlight the possible role of SA signaling in Tempranillo, especially when infected and pretreated with *Bs* PTA-271 (i.e., *PR1*). Curiously, the JA/ET-responsive gene *LOX9* was also highly upregulated in infected Tempranillo, while *LOX9* was severely downregulated in infected Tempranillo pretreated with single or both BCAs, as well as *PR2*, *PAL*, and *STS*. Opposite trends were observed in Chardonnay: (i) no prominent role of SA signaling in infected Chardonnay, especially when pretreated with *Bs* PTA-271; and (ii) *LOX9* was not so highly upregulated in infected Chardonnay (i.e., Ctl + Np), but *LOX9* was upregulated in infected Chardonnay pretreated with both BCAs, and *PR2*, *PAL*, and *STS* with *Ta* SC1 or both BCAs. These data could suggest the prominent role of JA/ET signaling in Chardonnay, especially when infected and pretreated with BCA. Interestingly, *PR2*, *PAL*, and *STS* are common defenses induced by each BCA against *Np*-Bt67 for the two cultivars, prossibly through two distinct signaling pathways.

### Direct Beneficial or Detrimental Interplays Between *Bacillus subtilis* PTA-271, *Trichoderma atroviride* SC1, and the Pathogen *Neofusicoccum parvum-*Bt67

**FIGURE 6:**
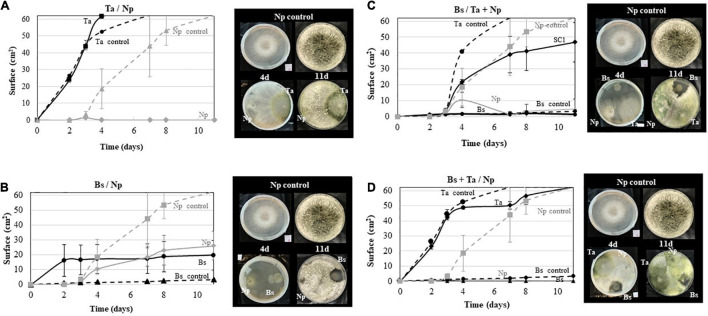
Antagonistic activity of *Bacillus subtilis* PTA-271 and *Trichoderma atroviride* SC1 against the *Neofusicoccum parvum* strain Bt67. **(A)** *T. atroviride* SC1 (Ta) was applied 48 h before *N. parvum* (Np) in the opposite sides of PDA plates. **(B)** *B. subtilis* PTA-271 (Bs) was applied 48 h before *N. parvum* (Np-Bt67) in the opposite sides of PDA plates. **(C)** *B. subtilis* PTA-271 (Bs) was applied 48 h before *N. parvum* (Np) and *T. atroviride* (Ta) at distinct areas of the PDA plates. **(D)** *B. subtilis* PTA-271 (Bs) and *T. atroviride* SC1 (Ta) were applied 48 h before *N. parvum* (*Np*) at distinct areas of the PDA plates. All plates were incubated at 28°C. Pictures of each plate condition were taken from day 1 to day 11 after the first inoculation. Photos on top are the control of *Np*-Bt67 and photos at the bottom indicate the confrontation assay at day 4 (left) and day 11 (right).

Regarding the *in vitro* tests with *Np*-Bt67 (Figure 6), results showed that *Bs* PTA-271 and *Ta* SC1 antagonize *Np*-Bt67 when plated 48 h before the pathogen. As shown in Figures 6A,B, the growth of *Np*-Bt67 was consistently reduced by *Ta* SC1 or *Bs* PTA-271 in dual confrontation compared to the control. However, while the growth of *Np*-Bt67 was completely repressed from day 3 by *Ta* SC1 (Figure 6A), it was half-repressed by *Bs* PTA-271 (Figure 6B), enabling the pathogen to grow consistently less than the control over the same time period. Thus, *Ta* SC1 antagonistic effect was stronger than that of *Bs* PTA-271, although only a fungistatic effect was observed between them (since when transplanted, the pathogen grows back).

In three-way confrontations (Figures 6C,D), the antagonistic effect of *Bs* PTA-271 against *Np*-Bt67 was still reinforced in the presence of *Ta* SC1, even applied 48 h later (Figure 6C). Such benefit was yet reinforced when the two BCAs were both applied 48 h before the pathogen, in which the growth of *Np*-Bt67 was close to 5 mm^2^ (Figure 6D). However, it should be noted that *Ta* SC1 did not grow as fast when applied 48 h after *Bs* PTA-271, since *Ta* SC1 slopes are not parallel but weaker in Figure 6C than in Figure 6D.

**FIGURE 7:**
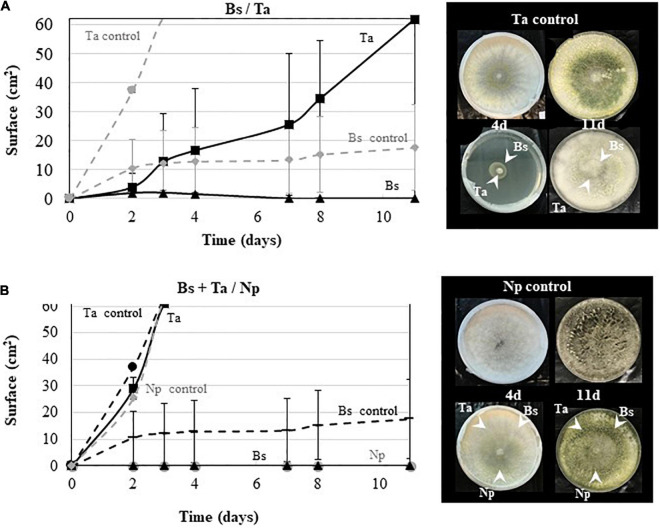
Antagonistic activity of *Bacillus subtilis* PTA-271 and *Trichoderma atroviride* SC1 against *Neofusicoccum parvum* Bt67. **(A)** *B. subtilis* PTA-271 (Bs) and *T. atroviride* SC1 (Ta) were applied simultaneously in the center of the PDA plates. **(B)** *B. subtilis* PTA-271 (Bs), *T. atroviride* SC1 (Ta), and *N. parvum* (Np) were applied simultaneously in the center of the PDA plates. All plates were incubated at 28°C. Pictures of each plate were taken from day 1 to day 11 after the first inoculation. Photos on top are SC1 control **(A)** and *Np*-Bt67 control **(B)**, and pictures at the bottom indicate the confrontation assay at day 4 (left) and day 11 (right).

To check the *Ta* SC1 capacity to keep its antagonistic effect when applied simultaneously with *Bs* PTA-271, dual confrontations were made between the two BCAs applied in the same area, with or without pathogen (Figure 7). As shown in Figure 7A, the growth of *Ta* SC1 was slowed down with *Bs* PTA-271, leading to a smaller *Ta* SC1 area than for the *Ta* SC1 control over the same time period. Interestingly, this detrimental effect of *Bs* PTA-271 on *Ta* SC1 disappeared in the three-way confrontation with the pathogen *Np*-Bt67 (Figure 7B), being all applied simultaneously at the same area. Thus, *Ta* SC1 and/or *Bs* PTA-271 may keep their strong antagonistic activity when facing a common adversary.

## Discussion

In search of an effective protective BCA combination against *N. parvum*, we investigated a potential BCA and a BCA-commercial product, each already described as protectors against GTDs on distinct cultivars: *B. subtilis* PTA-271 with Chardonnay (Trotel-Aziz et al., 2019) and *T. atroviride* SC1 with different cultivars (Pertot et al., 2016; Berbegal et al., 2020; Martínez-Diz et al., 2021a). This study assessed the combined impact of these two BCAs on two cultivars artificially infected or not with one pathogen. *N. parvum* Bt67 was used, as a very aggressive pathogen associated to BD. Our investigation focused on the capacity of single and combined BCAs to counteract BD symptoms on both cultivars. To compare the two BCAs, densities were aligned to the density optimized for *Bs* PTA-271. After looking for the protective capacity of BCA *in planta*, their modes of action leading to protection were further explored. Thus, we investigated whether these BCAs could affect pathogen growth *in vitro* and cultivar immunity upon infection *in planta*.

*Neofusicoccum parvum*-Bt67 caused BD symptoms on the rootlings of the two grapevine cultivars, as shoot full dieback, canker external necrosis, and shoot internal necrosis. Interestingly, the full dieback symptoms were more severe on Tempranillo than on Chardonnay (i.e., 37.5 and 28%, respectively, Figures 2D,G), suggesting a greater susceptibility to BD for Tempranillo than Chardonnay, as already reported by Luque et al. (2009) and Cobos et al. (2019). Although there is a lack of comparative data between cultivars, the distinct susceptibility of some cultivars to GTDs has already been reported (Travadon et al., 2013; Fontaine et al., 2016b; Chacon et al., 2020; Reveglia et al., 2021), even within a same cultivar from one region to another or depending on the vintage (Mimiague and Le Gall, 1994). However, in Chardonnay and Tempranillo rootlings pretreated with one or both BCAs before inoculation of the pathogen, BD symptoms were significantly reduced with *Bs* PTA-271 or *Ta* SC1, respectively. Grapevine effective protection against *Np-*Bt67 has already been reported with *Bs* PTA-271 on Chardonnay rootlings (Trotel-Aziz et al., 2019), and with *Ta* SC1 on Tempranillo in nursery and vineyards conditions (Berbegal et al., 2020; Blundell et al., 2021). Additionally, in our experimental conditions, Chardonnay seems to favor the beneficial effect of *Bs* PTA-271, while Tempranillo favors that of *Ta* SC1 beneficial effect, highlighting the relationship between cultivar response and BCA effect. Manter et al. (2010) suggested that the differences between cultivars may result from minor changes in the composition of their endophyte community, with *Trichoderma* species being among the most common endophytic fungal isolates from Tempranillo (González and Tello, 2011). Therefore, Tempranillo could be subjected to *Trichoderma*’s influence. Similarly, the efficiency of *Bs* PTA-271 toward Chardonnay may be explained by its origin of sampling from an established Chardonnay vineyard, screened from healthy vines (Trotel-Aziz et al., 2008). In combination and according to our experimental conditions, despite cited as compatible strains (Kumar, 2013), *Ta* SC1 + *Bs* PTA-271 are less protective against *Np*-Bt67 in Chardonnay than *Bs* PTA-271 alone. The authors reported that *Trichoderma* spp. can interfere the plant signaling networks and secrete an arsenal of degrading enzymes (i.e., proteases) and secondary metabolites (Tiwari and Verma, 2019; Alfiky and Weisskopf, 2021), suggesting that *Ta* SC1 may alter both *Bs* PTA-271 integrity and beneficial effects in Chardonnay. However, the application of both BCAs enabled to reach the highest level of Chardonnay defense gene expression upon pathogen challenge (see Figure 3A), and the highest protection in Tempranillo cultivar (see Figure 2G), highlighting that *Ta* SC1 on its own would not interfere with the beneficial effects of *Bs* PTA-271.

Beneficial effects of combined BCAs have yet been reported in different pathosystems (Yobo et al., 2011; Magnin-Robert et al., 2013), and our study reports for the first time the biocontrol potential of the combination of *Bs* PTA-271 and *Ta* SC1 against *Np*-Bt67 in Tempranillo. Our results showed that *Ta* SC1 efficiently protects Tempranillo, and this protection is still observed in rootlings pretreated with both BCAs (see Figure 2G). Thus, a significant benefit is observed when using both BCAs in Tempranillo, despite the fact that they could antagonize each other. In this respect, Leal et al. (2021) reported that the genome of *Bs* PTA-271 encodes for the synthesis of bacillaene, a polyketide already described to antagonize *Trichoderma* spp. (Caulier et al., 2019). Therefore, the positive contribution of *Bs* PTA-271 and *Ta* SC1 to Tempranillo protection against *Np*-Bt67 suggests a fine-tuned orchestrated cooperation of BCAs when facing adversity, as supported by the Figure 7 results and highlighted by Alfiky and Weisskopf (2021). However, the application of BCAs to rootlings was spatially separated.

Empowered with aggressive molecules, *Bacillus* spp. and *Trichoderma* spp. can possibly exert direct beneficial or detrimental interplays within the host’s microbiome, and especially on the other BCA and pathogens such as GTDs fungi (Di Marco et al., 2002; Kloepper et al., 2004; Ongena and Jacques, 2008; Haidar et al., 2016; Trotel-Aziz et al., 2019; Yacoub et al., 2020; Úrbez-Torres et al., 2020; Blundell et al., 2021). *In vitro* dual confrontation tests confirmed the antagonistic activity of *Bs* PTA-271 or *Ta* SC1 toward *Np-*Bt67 since each of them significantly inhibits the mycelium growth of *Np*-Bt67 (see Figures 6A,B). This also prompts us the idea that the not-yet-convincing protection assay of *Bs* PTA-271 and *Ta* SC1 in infected Chardonnay would not result from a detrimental effect of *Ta* SC1 on some putative direct effects of *Bs* PTA-271. Indeed, the strong direct antagonistic activity of *Bs* PTA-271 and *Ta* SC1 toward *Np*-Bt67 also operates when the two BCAs were applied both 48 h before the pathogen (see Figure 6D). This antagonistic benefit is in accordance with the significant synergy of the protection in Tempranillo by both BCAs (see Figure 2G), and it confirms the benefit of using them in combination in Tempranillo to optimize the direct fight against *Np*-Bt67. A similar outcome was reported by Alexander and Phin (2014) using an effective combination of *Bacillus* spp. and *Trichoderma* spp. against *Ganoderma* spp. Such direct effects of BCA against pathogens are important life traits to protect grapevine from BD, still deprived of effective curative treatments nowadays (Mondello et al., 2018). In nursery too, healthy mother plants require a control of their sanitary status (Pertot et al., 2016), eventually provided by an early inoculation of such beneficial BCA with a strong antagonistic activity toward pathogens.

Direct beneficial or detrimental interplays between BCAs also condition their capacity to live together in symbiosis, even *in planta* as part of the holobiont (Bettenfeld et al., 2020). Dual confrontation was thus also performed between *Bs* PTA-271 and *Ta* SC1. As expected, *Bs* PTA-271 antagonized *Ta* SC1 (see Figure 7A; Caulier et al., 2019; Leal et al., 2021). However, this detrimental effect of *Bs* PTA-271 on *Ta* SC1 disappears in a three-way confrontation with *Np*-Bt67, when they were all applied simultaneously at the same area (see Figure 7B). These data confirm that *Ta* SC1 and *Bs* PTA-271 can positively interact to better confine *Np*-Bt67 and can lead to a direct positive contribution of this combination to the protection of Tempranillo against *Np*-Bt67. However, such a direct positive contribution of combined BCAs did not operate on the infected Chardonnay rootlings. Our experimental conditions could have altered the ability of *Ta* SC1 to exert its direct fungistatic effect (applied once at 10^8^ CFU/ml with a paintbrush over a 5-mm^2^ area). This also strongly suggests that Chardonnay itself alters the fine-tuned orchestrated cooperation of BCAs, probably targeting the indirect *Ta* SC1 beneficial effect since BCAs are spatially separated. This prompts us to pursue our investigations further in order to decipher the indirect interactions driving to a beneficial outcome in grapevine control of *Np*-Bt67.

According to our previous works, a focus on grapevine systemic immunity was made by targeting six selected defense genes in leaves: the lipoxygenase *LOX9* involved in oxylipin synthesis and described as dependent to JA/ET; *PR1* described to be regulated by SA; the β-1,3-glucanase *PR2* described to be regulated by various phytohormones such as SA, JA, and ET; the glutathione-*S*-transferase *GST1* putatively involved in the detoxification process; the phenylalanine ammonia-lyase *PAL* catalyzing the first step in the phenylpropanoid pathway; and the stilbene synthase *STS* involved in the synthesis of phytoalexins (Trotel-Aziz et al., 2019). Interestingly, basal immunity results in a weak constitutive expression of the targeted defense genes in Chardonnay compared to Tempranillo (see Figure 3), despite the fact that literature described Chardonnay as less susceptible to BD than Tempranillo (Luque et al., 2009). Maybe our six targeted genes are not sufficiently exhaustive to presume at this preliminary stage of the susceptible versus tolerant status of both cultivars. However, looking at the specific red and white cultivar responses, the same studied genes are of interest: those specific to white grape cultivars include transcription factors from the ET pathway and lipid metabolism (e.g., lipoxygenase), while those specific to red grape cultivars are linked to the secondary metabolism in connection with the pathway of phenylpropanoids (e.g., PAL and its derivatives) and are expressed more strongly in the red cultivars, to distinguish them from the white ones (Lambert et al., 2012; Massonnet et al., 2017). Upon abiotic or biotic stress, other authors pointed out the highest synthesis of resveratrol in the most tolerant grapevine (Corso et al., 2015, Lakkis et al., 2019), but a gain of protection due to the BCA presence in susceptible cultivar (Lakkis et al., 2019). Considering that susceptible plants rather benefit from the help of BCA to induce their immunity, unlike resistant plants that already have high basal immunity, we examined the immunity induced by both cultivars studied.

In Tempranillo (see Figures 3B, 5 and Supplementary Figure 1B) under our experimental conditions, application of *Bs*-PTA-271 did not induce significant changes in plant defense responses compared to control, whether infected or not. Since Tempranillo basal immunity strongly upregulates *PR1* as a marker of SA-dependent defenses, we can speculate that high basal SA content might contribute to prevent the beneficial effect of *Bs* PTA-271 on Tempranillo immunity. Our previous study showed that *Bs* PTA-271 primed the expression of the plant JA/ET-dependent defenses in Chardonnay rootlings (Trotel-Aziz et al., 2019), whereas it is reported that early activation of SA signaling could antagonize the expression of these JA/ET-dependent defenses (Pieterse et al., 2012; Van der Does et al., 2013). In this sense, *Bs* PTA-271 did not provide protection in Tempranillo against *Np*-Bt67. These results show therefore that cultivars differing in their basal immunity can condition the success of a BCA protection toward a pathogen. In contrast, *Ta* SC1 alone or together with *Bs* PTA-271 acts as a priming stimulus for Tempranillo, but no post-priming was observed with *Ta* SC1 alone and with *Ta* SC1 + *Bs* PTA-271 upon pathogen challenge. Looking at the defenses induced by these two protective modalities (*Ta* SC1 and *Ta* SC1 + *Bs* PTA-271) against *Np*-Bt67: the SA-dependent defenses (i.e., *PR1*, *PAL*, and *STS*) were rather strongly decreased in protected plants (i.e., asymptomatic despite infected), while they were the highest in symptomatic plants (*Bs* PTA-271). Since Botryosphaeriaceae are known to specifically metabolize grapevine phytoalexins (Stempien et al., 2017), which benefits pathogen fitness, we could suggest that the SA stimulation of the phenylpropanoid pathway and derivatives would wrongly serve the plant. In the case of Tempranillo exposed to Botryosphaeriaceae, the high constitutive expression of SA-dependent defense genes could thus appear as a disadvantage, confirming that Tempranillo would be less tolerant than Chardonnay to BD, as already reported by Luque et al. (2009) and Cobos et al. (2019). Fortunately, in the Tempranillo pretreated with both BCAs, the expressions of genes *PR1*, *PAL*, and *STS* were repressed, and in the Tempranillo pretreated with *Ta* SC1 alone, the expression of the genes *PAL* and *STS* were repressed. Therefore, the beneficial effect of *Ta* SC1 on Tempranillo to control *Np*-Bt67 could result from a repressive effect on detrimental SA-dependent defenses. Complementary approaches are in progress to screen the induced key levers able to trigger ISR in the whole plant. Additionally, the *Ta* SC1 beneficial effect on Tempranillo could also result from a direct antagonism toward *Np*-Bt67 since it is applied in the same area as *Ta* SC1.

In non-infected Chardonnay rootlings (see Figures 3A, 4 and Supplementary Figure 1A), *Ta* SC1 shoot application did not induce any significant changes of the selected targeted responses of plant defenses in leaves, while *Bs* PTA-271 root application upregulated the *PR1* and *PR2* gene expression and the combined application induced the expression of almost all targeted genes at a higher level than *Bs* PTA-271 alone. *Bs* PTA-271, alone or together with *Ta* SC1, may thus act as a priming stimulus in Chardonnay. However, upon pathogen challenge (4 dpi, designed as a relevant sampling time point for such experiment), *Bs* PTA-271 did not prime any of the targeted defenses, probably due to low to medium pathogen pressure compared to that reported in Trotel-Aziz et al. (2019). However, *Bs* PTA-271 beneficial effect on Chardonnay is supported by the phenotype of the *Bs*-PTA-271 treated rootlings, showing a significant protection for Chardonnay against *Np*-Bt67 at 2.5 mpi. This contrasts with the detected non-benefit provided by the combined application of both BCAs at 2.5 mpi, despite the fact that defenses were primed at 4 dpi, possibly due to a very high level of reactive oxygen species (ROS) that could amplify the plant defenses. It is interesting to note that *GST1* (involved in the ROS detoxification process) is the only useful targeted gene that was weakly expressed in non-protected Chardonnay pre-treated with both BCAs and further infected with *N. parvum* (see Figure 3A). This may suggest that when a pathway with high induced defenses is not combined with sufficient ROS detoxification, a plant could potentially trigger many symptoms. In our experimental conditions, Chardonnay therefore favors *Bs* PTA-271’s beneficial effect, probably thanks to the different key levers including the induced grapevine immunity. Present at the root level, these results and those reported in Trotel-Aziz et al. (2019) suggest that systemic induced immunity conferred by *Bs* PTA-271 drove the plant ISR against *Np*-Bt67. Amazingly, the application of both BCAs enabled to reach the highest level of Chardonnay defense gene expression upon pathogen challenge (see Figure 3A), despite no protection at 2.5 mpi (see Figure 2D). Since *Ta* SC1 contributes to actively reducing the SA-dependent defenses in Tempranillo, one can hypothesize that *Ta* SC1 would also promote the *Bs* PTA-271 way of triggering immunity in Chardonnay. The authors also indicated that *Trichoderma* spp. may produce enzymes (i.e., ACC deaminase) able to shunt ET synthesis (Alfiky and Weisskopf, 2021), but in Tempranillo, the application of both BCAs enabled reaching the highest protection (see Figure 2G). Thus, it would be surprising that *Ta* SC1 on its own would succeed to interfere with the beneficial effects of *Bs* PTA-271 in Chardonnay. This opens the discussion of how the cultivar interacts with BCAs and the pathogen to condition the beneficial or detrimental outcome against *Np*-Bt67. Complementary approaches are already in progress to screen which are the induced key levers useful to control ISR in the whole plant.

## Conclusion

Altogether, our results provide evidence that grapevine susceptibility to BD is cultivar-dependent, as well as the BCA beneficial effects. *Bs* PTA-271 was confirmed as an effective protector for Chardonnay against *Np*-Bt67, and *Ta* SC1 was shown for the first time as a good protector for Tempranillo. This study also reports for the first time the biocontrol potential of the combination of *Bs* PTA-271 and *Ta* SC1 against *Np*-Bt67 in Tempranillo. This is a promising result for an improved efficiency of sustainable biological control in a proven context of lack of effective chemicals to manage GTDs.

Endowed with aggressive molecules, *Bs* PTA-271 and *Ta* SC1 can antagonize each other, but *Bs* PTA-271 inhibits *Np-*Bt67 development with a greater efficiency in a three-way confrontation. This beneficial BCA collaboration against *Np*-Bt67 still operates in Tempranillo and confirms the interest of using both BCAs in combination to optimize the direct fight against *Np*-Bt67. These results are of great interest for effective curative treatments to obtain healthy mother plants in the nursery and to control BD in vineyard. However, the direct beneficial effect of combined BCAs did not operate to protect Chardonnay, suggesting that Chardonnay itself probably alters the fine-tuned orchestrated cooperation of BCAs that drives such direct beneficial effect.

Plant systemic immunity was also affected by each BCA. Our findings suggest a common feature for the two cultivars: the defenses that are greatly diminished in BCA-protected plants appear to be those that are responsive to SA, in contrast to symptomatic plants. For Tempranillo, the high basal expression of SA-dependent defenses may thus explain the highest susceptibility to BD and also the ineffectiveness of *Bs* PTA-271 in our experimental conditions. Complementary approaches are underway to further investigate the responses of each cultivar to both *Bs* PTA-271 and *Ta* SC1 under controlled conditions and upon pathogen challenge.

## Data Availability Statement

The raw data supporting the conclusions of this article will be made available by the authors, without undue reservation.

## Author Contributions

CL, PT-A, and FF designed and planned the research. CL performed most of the experiments, analyzed the data, and wrote the manuscript with input and discussion from PT-A, FF, JA, and DG. JA and DG validated the planned research and provided expertise for all stages of this work. J-FG ensured the quality of the mother cuttings and greenhouse conditions. NR assured the quality of the qRT-PCR analyses and data. All authors contributed to writing and revising the manuscript.

## Conflict of Interest

The authors declare that the research was conducted in the absence of any commercial or financial relationships that could be construed as a potential conflict of interest.

## Publisher’s Note

All claims expressed in this article are solely those of the authors and do not necessarily represent those of their affiliated organizations, or those of the publisher, the editors and the reviewers. Any product that may be evaluated in this article, or claim that may be made by its manufacturer, is not guaranteed or endorsed by the publisher.

## Supplementary Material

Supplementary Figure 1 *Bacillus subtilis* PTA-271 and *Trichoderma atroviride* SC1 attenuates induce differential expression of defense-related genes in leaves of Chardonnay **(A)** and Tempranillo **(B)** rootlings before and after pathogen challenge. Twenty weeks old rootlings untreated or pretreated with PTA-271 or SC1 or both were further infected with sterile PDA plugs (Control, Bs, Ta, and Bs + Ta, respectively) or with mycelium plugs of *Np*-Bt67 (Ctl + Np, Bs + Np, Ta + Np, and Bs + Ta + Np, respectively). Transcript levels of defense-related genes monitored by qRT-PCR in plant leaf after 4 days of inoculation. Uninfected control of each cultivar was considered as a reference sample (1× expression level) for the same cultivar, and heatmaps represent changes in the transcript expression levels as indicated by the color shading. Data are the means from three representative replicates among five showing the same trends. Legends for genes are as in Figure 2.
