# Supplementary figures and images for: Cultivar Contributes to the Beneficial Effects of Bacillus subtilis PTA-271 and Trichoderma atroviride SC1 to Protect Grapevine Against Neofusicoccum parvum
Source: Front Microbiol. 2021 Oct 14;12:726132. doi: 10.3389/fmicb.2021.726132 (PMC8552030; doi:10.3389/fmicb.2021.726132)

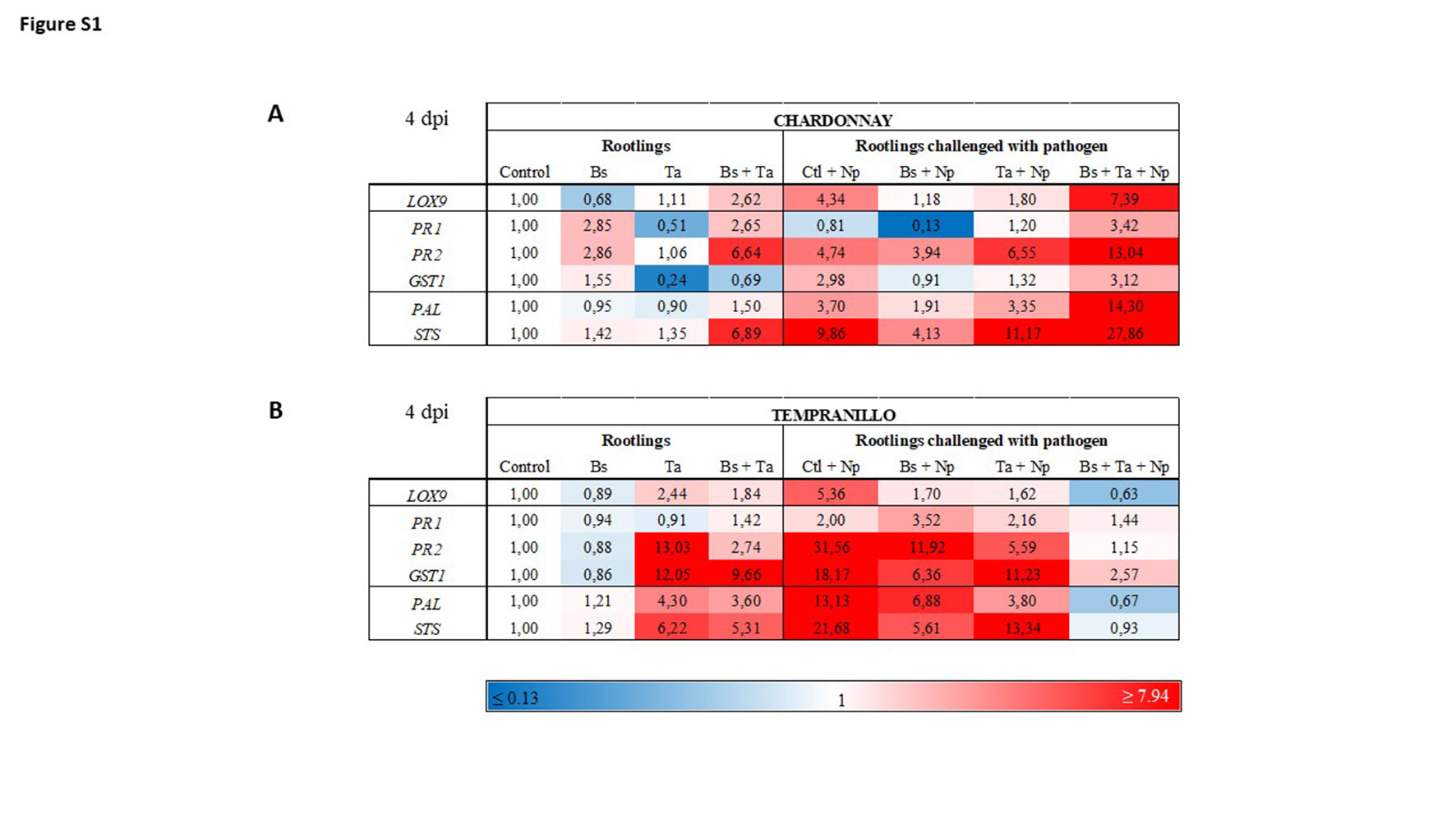

Supplement: Supplementary Figure 1 — Bacillus subtilis PTA-271 and Trichoderma atroviride SC1 attenuates induce differential expression of defense-related genes in leaves of Chardonnay (A) and Tempranillo (B) rootlings before and after pathogen challenge. Twenty weeks old rootlings untreated or pretreated with PTA-271 or SC1 or both were further infected with sterile PDA plugs (Control, Bs, Ta, and Bs + Ta, respectively) or with mycelium plugs of Np-Bt67 (Ctl + Np, Bs + Np, Ta + Np, and Bs + Ta + Np, respectively). Transcript levels of defense-related genes monitored by qRT-PCR in plant leaf after 4 days of inoculation. Uninfected control of each cultivar was considered as a reference sample (1× expression level) for the same cultivar, and heatmaps represent changes in the transcript expression levels as indicated by the color shading. Data are the means from three representative replicates among five showing the same trends. Legends for genes are as in Figure 2. [file Image_1.JPEG]
