# Supplementary material for: Cultivar Contributes to the Beneficial Effects of Bacillus subtilis PTA-271 and Trichoderma atroviride SC1 to Protect Grapevine Against Neofusicoccum parvum
Source: Front Microbiol. 2021 Oct 14;12:726132. doi: 10.3389/fmicb.2021.726132 (PMC8552030; doi:10.3389/fmicb.2021.726132)
Supplement: Supplementary file 2 [file Table_1.pdf]

TABLE 1: Primer sequences used for qRT-PCR analysis of defense-related genes (Trotel-Aziz et al. 2019).

Gene	Name	Accession number[1]	Forward Primer (5'-3')	Reverse primer (5'-3')	Annealing temperature (°C)	Amplicon size (bp)	Efficiency of primers pairs (%)
60RSP	60S ribosomal protein L18	XM_002270599[1]	ATCTACCTCAAGCTCCTAGTC	CAATCTTGTCCTCCTTTCCT	60	166	100.0
EF1	elongation factor 1-alpha	XM_002284888[1]	AACCAAAATATCCGGAGTAAAAGA	GAACTGGGTGCTTGATAGGC	60	164	100.0
LOX9	Lipoxygenase	NM_001281249[1]	CCCTTCTTGGCATCTCCCTTA	TGTTGTGTCCAGGGTCCATTC	60	101	90.0
PR1	pathogenesis-related protein 1	XM_002273752[1]	GGAGTCCATTAGCACTCCTTTG	CATAATTCTGGGCGTAGGCAG	60	168	90.0
PR2	Class I beta-1,3-glucanase	NM_001280967[1]	TCAATGGCTGCAATGGTGC	CGGTCGATGTTGCGAGATTTA	60	155	97.2
GST1	glutathione S-transferase	NM_001281248[1]	TGCATGGAGGAGGAGTTCGT	CAAGGCTATATCCCCATTTTCTTC	60	98	90.0
PAL	phenylalanine ammonia lyase	XM_003635637[1]	TCCTCCCGGAAAACAGCTG	TCCTCCAAATGCCTCAAATCA	60	101	92.9
STS	stilbene synthase	NM_001281117[1]	AGGAAGCAGCATTGAAGGCTC	TGCACCAGGCATTTCTACACC	60	101	94.3

[1] NCBI accession number.
